# A Survey on Data Storage and Information Discovery in the WSANs-Based Edge Computing Systems

**DOI:** 10.3390/s18020546

**Published:** 2018-02-10

**Authors:** Xingpo Ma, Junbin Liang, Renping Liu, Wei Ni, Yin Li, Ran Li, Wenpeng Ma, Chuanda Qi

**Affiliations:** 1 School of Computer and Information Technology, Xinyang Normal University, Xinyang 464000, Henan, China; yunfeiyangli@gmail.com (Y.L.); liran@xynu.edu.cn (R.L.); markwinpe@gmail.com (W.M.); qichuanda@gmail.com (C.Q.); 2 School of Computer and Electronic Information, Guangxi University, Nanning 530004, China; liangjb@gxu.edu.cn; 3 Global Big Data Technologies Centre, University of Technology Sydney, Ultimo 2007, Australia; RenPing.Liu@uts.edu.au; 4 Data61, CSIRO, Sydney NSW 1466, Australia; Wei.Ni@data61.csiro.au

**Keywords:** Internet of Things, Edge computing, WSANs, data storage, information discovery

## Abstract

In the post-Cloud era, the proliferation of Internet of Things (IoT) has pushed the horizon of Edge computing, which is a new computing paradigm with data processed at the edge of the network. As the important systems of Edge computing, wireless sensor and actuator networks (WSANs) play an important role in collecting and processing the sensing data from the surrounding environment as well as taking actions on the events happening in the environment. In WSANs, in-network data storage and information discovery schemes with high energy efficiency, high load balance and low latency are needed because of the limited resources of the sensor nodes and the real-time requirement of some specific applications, such as putting out a big fire in a forest. In this article, the existing schemes of WSANs on data storage and information discovery are surveyed with detailed analysis on their advancements and shortcomings, and possible solutions are proposed on how to achieve high efficiency, good load balance, and perfect real-time performances at the same time, hoping that it can provide a good reference for the future research of the WSANs-based Edge computing systems.

## 1. Introduction

With the fast development of Internet of Things (IoT) [1] and the coming fifth generation mobile communication systems (5G) [2,3], we are now arriving in the post-Cloud era, where a large quality of data are generated by things and many applications will also be deployed at the edge of the network to consume these data. Because data are produced at the edge of the network increasingly, it would also be more efficient to process the data at the edge, which leads to a novel computing paradigm, namely Edge computing [4,5].

In recent years, an important network paradigm of Edge computing, namely Wireless Sensor and Actuator Networks (WSANs) [6,7], has entered a stage of rapid development, and more and more WSANs-based Edge computing systems have been used in a lot of applications, such as new energy resources [8], industrial automation [9], smart agriculture [10], intelligent transportation [11], building automation [12], and environment monitoring and protection [13,14]. Unlike Wireless Sensor Networks (WSNs) [15,16,17], which mainly contain sensor nodes organized in a wireless and Ad-hoc manner, WSANs are heterogeneous networks and consist of not only sensor nodes but also actuators. The sensor nodes in WSANs take the similar tasks as they are in WSNs, such as data collection, while the actuators are responsible for taking actions on the events happening in the monitored field as well as processing and storing the data collected by the sensor nodes. Generally speaking, the actuators have much more resources, such as the storage space and the energy, much stronger computation capability and longer communication radius than the sensor nodes. Thus, protocols and schemes should be designed specifically for WSANs rather than immigrating them directly from WSNs. 

In this paper, we focus on the data storage and information discovery technologies, which are the core technologies of the WSANs-based Edge computing systems, in WSANs and conduct a survey of the existing related schemes. In summary, the main contributions of this paper are listed as follows:

1. We first summarize the data-storage and information-discovery schemes proposed for WSNs, and analyze why those schemes are not fit for the WSANs-based Edge computing systems. We do this because there are many similarities between the WSANs-based Edge computing systems and the WSNs-based systems.
2. The existing schemes proposed for WSANs on data storage and information discovery are surveyed and compared with each other, and detail analysis on their advancements and shortcomings is also presented.
3. Possible solutions are given on how to achieve high efficiency, high load balance, and perfect real-time performances at the same time for data storage and information discovery in the WSANs-based Edge computing systems.

The remainder of this paper is organized as follows: in Section 2, we summarize the existing related schemes for WSNs, and analyze why they are not fit for the WSANs-based Edge computing systems. In Section 3, we survey the schemes on data storage and information discovery proposed for WSANs, and discuss their advancements and shortcomings. In Section 4, possible solutions to achieving the aforementioned multi-objectives in the WSANs-based Edge computing systems are proposed. In Section 5, we conclude the paper.

## 2. Analyzing Whether the Data-Storage and Information-Discovery Schemes Proposed for WSNs Fit for WSANs

Before surveying the data-storage and information-discovery schemes for the WSANs-based Edge computing systems, we first categorize schemes proposed for WSNs because there are many commonalities between WSNs and WSANs. In WSNs, the problem of data storage and information discovery has been studied for many years. In a nutshell, data are stored in WSNs mainly based on the following three models: (1) data are stored among the sensor nodes in a distributed manner [18,19,20,21,22,23,24,25,26,27,28]; (2) data are stored at the static Sink node/nodes intensively [29,30,31,32,33]; (3) data are collected and stored at some mobile elements [34,35,36,37,38,39,40,41], such as mobile Sinks. For the first model, queries should be launched across the sensor nodes to search for and discover the needed information; for the second and third models, the needed information can be discovered directly at the static Sink node/nodes or the mobile elements.

Although these models work well in WSNs, they may not be fit for WSANs. First of all, a storage model that allows all the sensing data to be stored among the sensor nodes may not be suitable for WSANs. Unlike WSNs, which mainly consist of sensor nodes, WSANs are heterogeneous and composed of not only the sensor nodes but also the actuators. The actuators have more resources and longer communication ranges than the sensor nodes. Thus, if all data are stored at sensor nodes, there can be a waste of the resources and the capabilities of the actuators. Moreover, the data-storage and information-discovery schemes based on this model in WSNs have not taken into account the problem of coordination between the sensor nodes and the actuators, which only exists in WSANs. Secondly, the centralized storage model, which relies on static Sink nodes, is not applicable to WSANs. The reason is because: on the one hand, the storage schemes proposed for WSNs based on this model still lack negotiation and the coordination mechanisms between the sensor nodes and the actuators; on the other hand, the actuators in WSANs are usually mobile and can respond to the events that may happen in other regions. Finally, the data collection model utilizing mobile Sinks or elements are not unsuitable for WSANs either. In WSANs, the actuators have to move to the locations where events occur to take some actions. Because the events may happen anywhere at any time, the destinations for the actuators to move to can be random, and the frequencies for actuators to move are also random. Moreover, because a quick response is required in WSANs to respond to the events occurring in the monitored field, the actuators need to move to the destinations directly as fast as possible. Thus, it is impossible to design the moving paths and the time duration to stay in each of the locations for the actuators to collect data, as the mobile elements typically do in WSNs.

From the analysis presented above, we can see that it is non-trivial to migrate the data-storage and information-discovery schemes developed for WSNs directly to WSANs, and specially designed novel schemes need to be developed for the WSANs-based Edge computing systems.

## 3. Analysis of the Data-Storage and Information-Discovery Schemes Proposed for WSANs

To the best of our knowledge, the WSANs-based Edge computing systems on data storage and information discovery are emerging research areas, and not much research has to date been carried out [42,43,44,45,46,47,48,49,50,51,52,53,54]. Those existing schemes mainly follow two basic models: the query-driven model and the event-driven model. In the following, we describe these two models separately and analyze the related schemes based on them.

### 3.1. Schemes Based on the Query-Driven Model

As for the query-driven model, sensing data generated by the sensor nodes are stored in a distributed manner in WSANs, and queries are launched across the network to search the information that the consumers (sensor nodes, actuators, or other network users) are interested in. The typical storage method based on this model is data-centric storage [42], where a distributed storage system is constructed according to the data or event type. In the distributed storage system, each event type has a mapping node, also known as rendezvous node or home node. Because of the limited storage capacity of a mere sensor node, the mapping node may have one or more replicas in the storage system. When data are generated by the sensor nodes, the data will be sent to the mapping node or its replicas and stored according to the type of the data. A consumer interested in retrieving the data needs to launch and send a query to the mapping node or its replicas according to the interesting data type.

**Figure 1:**
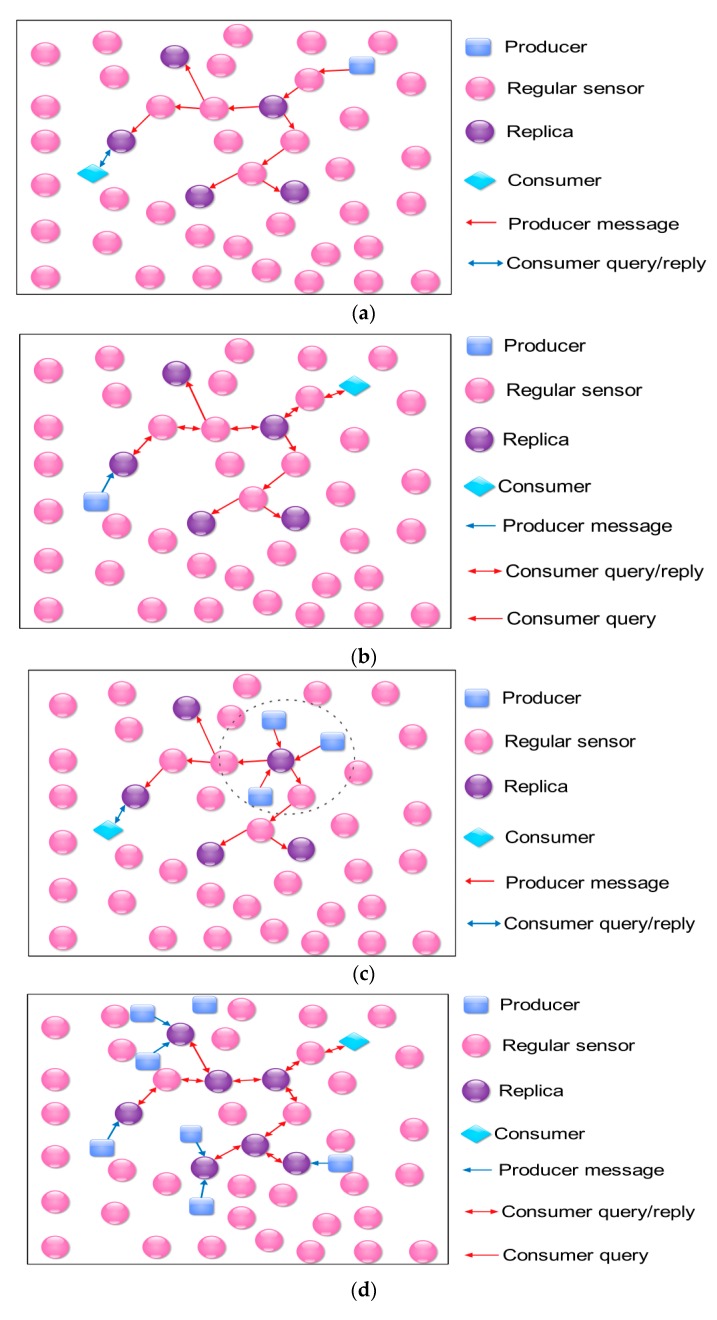
Data storage and information discovery in the four application profiles: (**a**) consumption traffic dominates production traffic with no data aggregation; (**b**) production traffic dominates consumption traffic with no data aggregation; (**c**) consumption traffic dominates production traffic with data aggregation; (**d**) production traffic dominates consumption traffic with data aggregation.

In 2011, Cuevas et al. analyzed several data-centric-storage and information-discovery schemes and found that the schemes using more than one rendezvous node perform much better than those just using a single rendezvous node for each data type in terms of minimizing the overall network traffic [42]. They classify the applications of data storage and information discovery into four profiles according to the taxonomy whether the data are aggregated or not and whether the consumption traffic dominates the production traffic or the other way around: (1) the consumption traffic dominates the production one with no data aggregation; (2) the production traffic dominates the consumption one with no data aggregation; (3) the consumption traffic dominates the production one with data aggregation; and (4) the production traffic dominates the consumption one with data aggregation. For each profile, they design a data-storage and information-discovery scheme. Specifically, in the first application profile, event data that are generated by any producer are first sent to the closest replica of the producer to get stored, and then the replica sends the copies of the event data to the remaining replicas. Any consumer just needs to send a query to the nearest replica to retrieve the data that it is interested in; in the second application profile, any producer stores its own data just at the closest replica. To discover the interesting information, any consumer first sends a query to its closest replica, and then the query will be forwarded in turn to the remaining replicas; in the third application profile, the way to store the data and discover the information is similar to that in the first profile, and the mere difference is that the event data in the third profile should be aggregated at the replicas before being forwarded to the left ones rather than forwarded directly; the last application profile is similar to the second one, the difference is that, after a consumer forwards a query along a data replication tree, which is rooted at the closest replica, all the retrieved data should be aggregated at all the replicas they pass in the replication tree. The mechanisms of data storage and information discovery in the four application profiles are illustrated in Figure 1.

Although the authors in [42] have considered many scenarios to make the data-storage and information-discovery schemes as perfect as possible and they even have designed four analysis models to compute the optimal number of replicas corresponding to the four application profiles (Figure 1), there is something more they need to do because they did not consider the update of the replicas. Generally speaking, the loads of the replicas are much heavier than other normal sensor nodes. If the replicas cannot exchange roles with the normal sensor nodes, they will die much faster and the network lifetime will be shrunk greatly. Moreover, if the replicas never have been changed, the data stored on the replicas will not last long, and they will be overwritten within a short time period because of the limited storage capacity of the replicas.

In 2014, to support long-term storage as well as prolonging the lifetime of WSANs, Angel Cuevas et al. proposed a novel data-centric storage framework [44], in which the rendezvous nodes are updated periodically based on periods of fixed duration called epochs so that it is possible to perform temporal queries to previous rendezvous nodes in order to discovery information from the past [44]. The significant contribution in [44] is that it presents a model to compute the optimal number of replicas that can maximize the data availability. Specifically, suppose *r* sensor nodes out of *N* nodes are selected out as the replicas, then the optimal value of *r* is the one to minimize the probability *P*(*A_i_*(0, *t*] > *S*, ∀*i* = 1, 2, …, *r*), which is shown in Equation (1) [44], assuming that *N* >> *r*:

(1) $$P(A_{i}(0,t]>S,\;\forall i=1,2,\ldots )={\left(1-\sum_{i=1}^{S}\left(\begin{array}{c} t\\ i \end{array}\right){\left(\frac{r}{N}\right)}^{i}{\left(1-\frac{r}{N}\right)}^{t-i}\right)}^{r}.$$

In Label (1), *A_i_*(0, *t*] denotes the times that the *i^th^* node is selected as a replica after epoch 0 and before epoch *t*, and *S* symbolizes the ratio of the number of events for which a replica can store in its storage space to the number of events a sensor node needs to store in an epoch.

From our point of view, the schemes mentioned above are generally unsuitable for WSANs because they are similar with those proposed for WSNs and can be seen as a straightforward extension of WSNs to WSANs. Those schemes cannot effectively utilize the rich resources of the actuators, and they do not consider the mobility feature of the actuators either. In fact, because of the mobility of the actuators, it is challenging to take actuators as the rendezvous nodes traditional data-centric storage schemes should not be straightforwardly applied. Moreover, using such a data-centric storage model can hardly achieve real-time information discovery because event data are not sent directly to the actuators.

### 3.2. Schemes Based on the Event-Driven Model

In the schemes with event-driven models, when sensor nodes detect the events happing in the monitored field, they send them directly to actuators. Thus, actuators can acquire the event data without launching queries, and the real-time information discovery can be achieved. In this model, the challenging problems include how to ensure the real-time, reliable, secure and lightweight routing algorithms from the sensor nodes to the actuators [45,46,47,48,49], how to improve the coordination among the sensor nodes and the actuators [50], as well as how to execute tasks efficiently for the actuators [51,52,53].

**Figure 2:**
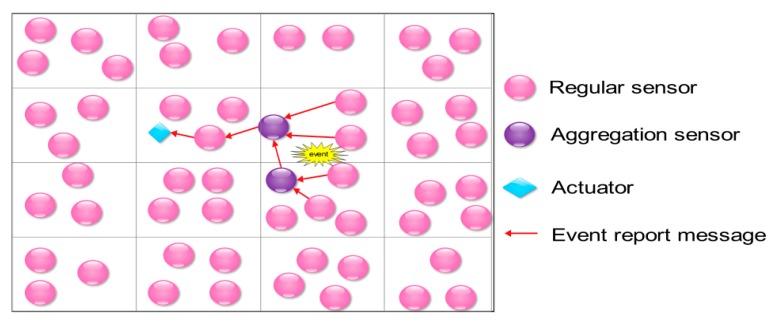
Data aggregation and transmission in the framework proposed in [45].

In 2010, to improve the reliability and the real-time performances of the event-data transmission from the sensor nodes to the actuators, Dr. Edith Ngai proposed a delay-aware reliable event-reporting framework for WSANs [45]. The overall reliability index ℝ used in [45] can be formalized as

(2) $$\mathbb{R}=\sum_{\forall e}\left(\frac{Imp\left(e\right)}{\sum_{\forall e}Imp\left(e\right)}\times r_{e}\right),$$

with the condition that the end-to-end delay of data report is smaller than or equal to the latency bound of reporting an event *e*. In Label (2), *Imp*(*e*) symbolizes the importance degree of the event *e*, and *r_e_* denotes the reliability index of the event *e*, where *r_e_* can also be comprehended as the proportion of the data reports that arrive at an actuator within a given delay bound and without data-aggregation and transmission failure. In this framework, the sensor field is divided into grids. In each grid, a random sensor node is in turn selected out as an aggregation node, it aggregates the event data from all the other sensor nodes in its own grid and then sends them to another aggregation node in another grid to get further aggregation. Finally, the aggregation result will be sent to the actuator by a reporter, which is selected from the aggregation nodes. This procedure is illustrated in Figure 2.

**Figure 3:**
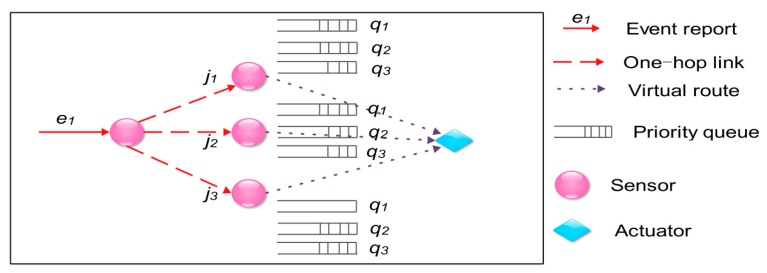
Route selection utilizing the priority queue model (priority: *q*_1_ > *q*_2_ > *q*_3_).

The core model of the framework proposed in [45] is the routing and transmission protocol from the reporter to the actuator. To make as many reports as possible reach the actuator within the latency bound and the reports that have higher importance levels reach the actuator with less latency, the protocol utilizes a priority queue model in each sensor node. In other words, each sensor node has several queues, each of which corresponds to an importance level, in its cache. Packets with higher importance levels will be placed in the corresponding higher-level queues, and will be transmitted prior to the ones with lower importance levels. Moreover, the priority queue model is also used to determine the route selection. Take Figure 3 as an example. When node *i* receives an event report *e_i_*, it will deliver it to node *j*_3_ because the queue with highest importance level in *j*_3_ is empty so that *e_i_* can be transmitted with less latency by *j*_3_.

To the best of our knowledge, the framework in [45] is the first to study WSANs from the data-importance point of view. However, this framework can only work well when the actuators are static. In the scenarios where the actuators can move randomly, the framework does not include a method on how to search the nearest actuators for the sensor nodes. In fact, due to the duty cycle of sensor nodes and the actor mobility, it is a challenging issue to forward the data from sensor nodes to mobile actors effectively with low delay.

**Figure 4:**
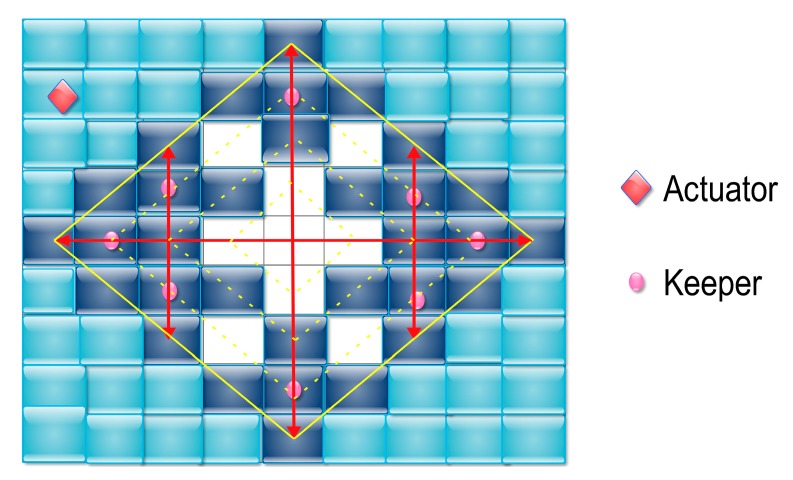
The ballooning strategy (the keepers are the sensor nodes who received the searching messages generated by the source; the white grids represent the cleared grids, the deep blue grids are the contaminated grids, and the light blue grids represent the clearing grids) [54].

In 2011, Xu et al. proposed a location-searching strategy namely ballooning [54] to find out the latest locations of the mobile actuators. In the network model presented in [54], the network field is divided into grids, and all the grids are classified into three categories: the cleared grids, the contaminated grids, and the clearing grids. By making the clearing grids form a closed source-centered balloon that packages all the cleared grids, ballooning achieves the aim that any contaminated grid is not adjacent to any cleared one, since they are separated by the clearing grids inside the balloon. As the closed balloon grows larger and larger, the latest actuator will be discovered once it is covered by the clearing grids. The ballooning strategy is illustrated in Figure 4.

Although using the ballooning strategy can ensure that the actuators can be discovered within a latency bound, there is still big space to improve its energy efficiency because of the inflation of the balloon in many directions. The worst case is that the actuator is discovered at the boundary of the network field so that the discovery message has to be broadcasted almost every node in the network during the inflation of the balloon. Moreover, in ballooning, no method is presented to solve the problem how to make the balloon stop inflating at other directions when the latest actuator is discovered at one direction.

In 2012, Xu et al. proposed another location-searching protocol, namely MLS (Mobility Location Service) [55]. In MLS, the network field is also divided into grids as in [54], and the only actuator moves in a Random Waypoint Model [56] with no pause time (in the Random Waypoint Model, an actuator repeats to do the following two steps: (1) choose a destination randomly; and (2) move to the destination with a constant speed *v* along a straight line.). Each time when the actuator reach a destination (*x*_0_, *y*_0_), it disseminates an update package, which includes: (1) the current timestamp *t*_0_; (2) the current location (*x*_0_, *y*_0_); (3) the destination (*x*_1_, *y*_1_); and (4) the moving time *τ*, to all the nodes, which act as the location servers during the moving time *τ*, in the grids of the same column as the actuator is currently located in. The moving time *τ* can be calculated as follows [55]:

(3) $$\tau =\frac{\sqrt{{\left(x_{1}-x_{0}\right)}^{2}+{\left(y_{1}-y_{0}\right)}^{2}}}{v}.$$

**Figure 5:**
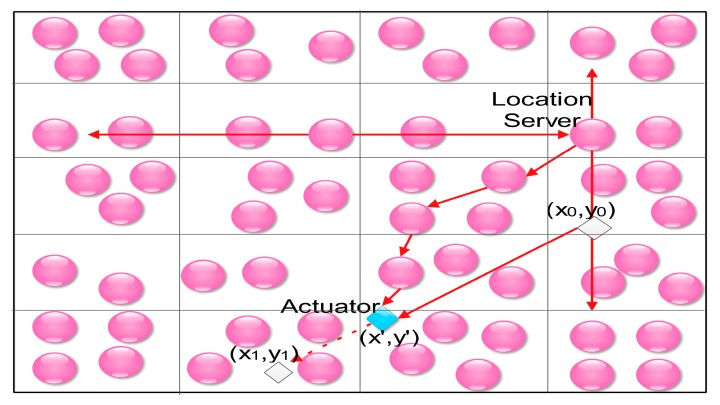
MLS: mobility strategy sharing location service [55].

When a sensor node (the source) detects an event, it forwards the event report at both west and east directions. One of the location servers must receive the event report finally because of the intersection of the rows and the columns. Then, the location server who receives the event report estimates the current location (*x*′, *y*′) of the actuator according to Equation (4) [55], where *t* is the timestamp when the location server who received the event report begins to estimate the location of the actuator, and then forwards the event report to the actuator using geographic routing protocol [57]. This procedure is shown in Figure 5:

(4) $$\{\begin{array}{c} x^{\prime }=x_{0}+\frac{t-t_{0}}{\tau }\times \left(x_{1}-x_{0}\;\right)\\ y^{\prime }=y_{0}+\frac{t-t_{0}}{\tau }\times \left(y_{1}-y_{0}\;\right) \end{array}.$$

Although the simulation results in [55] show that MLS performs better than some of the existing schemes about location services in WSANs on energy efficiency and scalability, it cannot cover up the obvious drawback of MLS that it requires perfect time synchronization, which is hard to be achieved in WSANs.

From the above-mentioned schemes, we can find that it is very important to find out an energy-efficient and delay-bounded strategy to deliver the events detected by the sensor nodes to the actuators or the Sink (for some WSANs which include the Sink) during the procedure of data storage and information discovery in WSANs. In fact, to achieve a QoS (Quality of service)-support routing, another metric should also be considered: the delivery ratio. In 2013, Mustafa et al. proposed a dynamic-interest-based lightweight routing protocol named LRP-QS (Lightweight Routing Protocol with QoS Support) [47]. In LRP-QS, the sensor nodes can evaluate the importance rankings of the event reports corresponding to the interests, which are actually the types of the event data and disseminated from the Sink to the actuators and the sensor nodes, locally according to the variable quality of the event data of each interest. Specifically, the interest with higher value fluctuation has higher importance ranking than those with lower value fluctuation for a given time period, and the ones with higher importance rankings will be allocated more resources to ensure their delivery quality. The simulation results in [47] show that LRP-QS can achieve a higher packet delivery ratio and a lower memory consumption than the existing state-of-the-art protocols.

To a certain degree, Ref. [47] shows that differentiating data according to their importance ranking can directly affect the QoS performance of the routing protocols, and consequently affect the performances of data storage and information discovery of WSANs in terms of time and energy efficiency.

**Figure 6:**
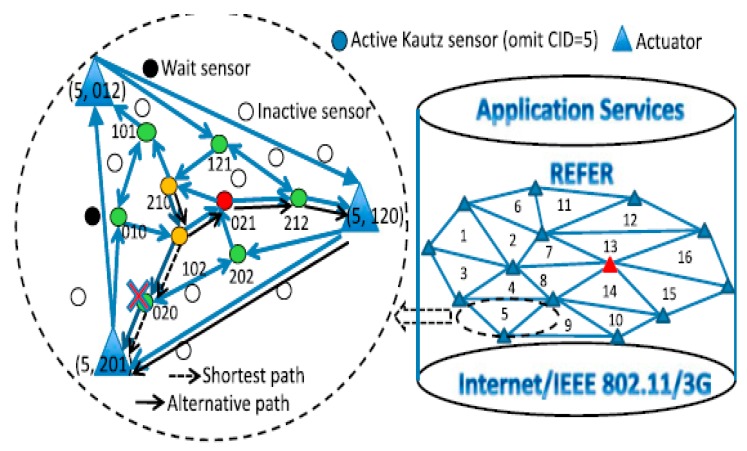
The architecture of the REFER system [48] (source node 210 wants to send an event report to node 201, and it sends the report along the route 210→102→020→201 where “→” denotes an unidirectional link. In the case that node 020 is broken, node 102 can independently find out an alternative route, namely 102→021→212→120→201, to route the report to the destination without requiring the source node to retransmit the report).

In 2016, to enable sensing data to reach actuators reliably and efficiently, Shen et al. proposed a Kautz-based REal-time, Fault-tolerant and EneRgy-efficient WSAN system(REFER) [48]. REFER divides a WSAN field into cells, and embeds Kautz graphs into the physical topology of a WSAN in each cell. Then, it connects the Kautz graphs in every cell using Distributed Hash Table (DHT) for high scalability. The architecture of the REFER system is shown in Figure 6. In this system, communications can be classified into two types: intra-cell communications and inter-cell communications. A sensor node that detects an event firstly transmits the event report to one of the actuators in its own cell using inter-cell communications, and then the event report is transmitted by the actuators using intra-cell communications. After studying the routing paths in the Kautz graph-theoretically, an efficient fault-tolerant routing protocol, an example of which is shown in Figure 6, was also proposed in [48] based on the Kautz graph.

The simulation results in [48] show that REFER can outperform many other existing WSAN systems in terms of energy efficiency, fault-tolerance, real-time communication, and scalability. However, the cell division and the maintaining of the Kautz-graph-based topology in each cell all depend on the locations of the actuators. If the actuators move randomly and frequently, it would cost much energy to maintain the Kautz-graph-based topology. Thus, REFER is much more suitable for the scenarios where actuators are static.

### 3.3. Comprehensive Comparison and Analysis

From the description and the analysis presented above, we can see that the existing schemes based on the query-driven model mainly store the sensing data in a distributed way among the sensor nodes, and it can hardly achieve the real-time information discovery. As for the existing schemes based on the event-driven model, although sending the data directly to the actuators to get stored can shrink the delay of discovering the event information, the energy consumption on updating the latest locations of the actuators across the network should attract our attention if the actuators move frequently. Moreover, if the amount of the sensing data is large, sending them all to the mobile actuators, which may not be located at the optimal storage locations because of carrying out the tasks, will not be energy efficient.

To illustrate the performances of the above-mentioned WSANs-based Edge computing systems on different metrics clearly, we make a tabular presentation in Table 1.

## 4. Possible Solutions to Achieve the Multi-Objectives on Data Storage and Information Discovery in the WSANs-Based Edge Computing Systems

In this section, we discuss potential technologies to achieve multi-objectives, such as high energy efficiency, high load balance and perfect real-time performance for data storage and information discovery in the WSANs-based Edge computing systems.

First of all, collaborative mechanisms for data storage and information discovery in WSANs should be researched and utilized. Both sensor nodes and actuators in WSANs should undertake part of the tasks of data storage and information discovery to improve the utilization ratio of the storage capacity of all types of nodes in WSANs and the load balance of WSANs. From the analysis presented in Section 3, we notice that the existing data storage schemes in WSANs either store all data on sensor nodes or store them all on actuators. It is hard for those schemes to achieve the above-mentioned multi-objectives in WSANs, the reasons of which are described as following: (1) consider the case that all data are stored on the actuators in WSANs. In this case, if sensor nodes send their sensing data directly as soon as the data are generated, each actuator must broadcast its latest location every time when it moves to a new place so that sensor nodes are able to know where to send their data. Moreover, because the events may occur at any time and any place in the network field and actuators must move to the places where the events happen to deal with them, it is impossible for the actuators to stay at the optimal storage locations all the time. Thus, it is hard to achieve high energy efficiency on data storage if all data are sent to the actuators and stored as soon as they are generated. It is straightforward to propose to let the actuators collect the data just like the data-collection schemes based on mobile elements in WSNs. Of course, this is energy-efficient, but its real-time performance would be compromised because it takes a lot of time for the actuators to move and collect the data of all the sensor nodes, especially when the network fields are large; (2) consider the case that all data are stored on sensor nodes in a distributed way. In this case, the actuators have to launch queries to search for the data that they are interested in. On one side, if the query frequencies of the actuators are low, it is impossible to achieve real-time information discovery. On the other side, if the query frequencies of the actuators are high, the energy efficiency will be low, especially when the events happen infrequently.

Considering the heterogeneous character of WSANs, to make the sensor nodes and the actuators collaborate efficiently on data storage and information discovery, a hierarchical storage model should be more suitable for WSANs. In other words, the sensor nodes are at one level while the actuators are at another level.

Secondly, task allocation for the actuators should tend to improve the load-balance performance of the schemes on data storage and information discovery in WSANs. When an event is detected in a WSAN, existing schemes on task allocation require the actuator, which is the closest to the place where the event occurs, to move to the place and deal with the event. As an alternative, choosing actuators randomly to deal with the events may be a better choice from the load-balance point of view. In this way, the distribution of the actuators can be adjusted adaptively according to the data generating rates of the sensor nodes. The final adjusting result should be that the regions with more events happening will attract more actuators. Thus, there will be more nodes with better storage capacity to share the storage load in the regions where data-generating rates are high. Moreover, for the sensor nodes that need to store their data on the actuators, choosing actuators randomly to get their data stored will also improve the load balance of the sensor nodes themselves because they should send the data along different routes to reach different actuators in different places randomly.

Finally, to achieve the aforementioned multi-objectives for data storage and information discovery, the data generated in WSANs should also be studied. By observation, we find that the data generated in WSANs are different in importance. For example, the outliers are more important than the normal data because the emergency of the outliers implies the abnormal events; the data with small emerging probabilities are more important than those with bigger emerging probabilities because the former contains much more information; the data that meet the interests of the users are more important than those do not meet the interests of the users because only the users are concerned about the data that they are interested in. Moreover, we also find that data with different importance levels (or priorities) have different characteristics and requirements for WSANs. For instance, the data with higher importance levels are generally generated with lower rates, and the total amount of them is relatively small. They have much higher requirements on the real-time performance of the data-storage and information-discovery schemes in WSANs. For the data with lower importance levels, their generating rates are relatively much higher, and they require the data-storage and information-discovery schemes to perform much better on energy efficiency and load balance. As for the data that are not important at all or even invalid [58], the sensor nodes can even drop them to save the resources. Thus, based on the hierarchical data storage model, the future proposed schemes that will involve different data-storage and information discovery mechanisms for the data with different importance degrees should be possible solutions for achieving the aforementioned multi-objectives in the WSANs-based Edge computing systems.

## 5. Conclusions

The WSANs-based Edge computing systems are meeting their fast developing opportunities in the post-Cloud era, and are used in more and more applications. As one of the most important technologies in the systems, the data-storage and information-discovery technology is surveyed in this paper. By analyzing existing works, we find that the existing schemes of WSNs are not suitable for WSANs because of their different network architectures and characteristics. Moreover, the existing schemes of WSANs still have many shortcomings, one of which is that they cannot achieve multi-objectives including high energy efficiency, high load balance and perfect real-time performance at the same time. Possible solutions, as proposed in this paper, to overcome the shortcomings of the existing schemes in WSANs are based on our observation and analysis, and we suggest they should be utilized during the future research on data storage and information discovery in the WSANs-based Edge computing systems.

Moreover, according to our surveys, few existing schemes of data storage and information discovery have considered the related security issues, such as how to preserve the integrity of the data stored on the actuators and how to achieve efficient mutual authentication among the nodes at different levels of WSANs in order to ensure that the important information in WSANs is only able to be discovered by the trustworthy nodes. Thus, joining the security consideration should be another future research direction of data storage and information discovery in the WSANs-based Edge computing systems.

Finally, existing schemes of data storage and information discovery in the WSANs-based Edge computing systems mainly consider the case that all sensor nodes are static. In the coming 5G era, there will be a lot of data which are generated by sensors on mobile devices [59]. Thus, how to achieve the above-mentioned multi-objectives for the WSANs-based Edge computing systems on data storage and information discovery in mobile scenarios is another open issue.

## Figures and Tables

**Table 1:** Performances of different WSANs-based Edge computing systems.

Systems	Energy Efficiency	Real-Time Support	Load Balance	Fault Tolerance	Actuator Movement Support
Traditional data-centric WSANs [42]	high	×	bad	bad	√
Long-term storage WSANs [44]	high	×	good	good	√
Delay-aware WSANs [45]	high	√	good	good	×
Ballooning [54]	low	√	good	good	√
MLS [55]	low	√	good	good	√
LRP-QS [47]	high	√	good	good	×
REFER (REal-time, Fault-tolerant and EneRgy-efficient WSAN) [48]	high	√	good	good	×

## References

[1] Al-Fuqaha A., Guizani M.M., Mohammadi M.M., Mohammed A., Moussa A. (2015). Internet of things: A survey on enabling technologies, protocols, and applications. IEEE Commun. Surv. Tutor..

[2] Chen S., Zhao J. (2014). The requirements, challenges, and technologies for 5G of terrestrial mobile telecommunication. IEEE Commun. Mag..

[3] Han T., Ge X., Wang L., Kwak K.S., Han Y., Liu X. (2017). 5G converged cell-less communications in smart cities. IEEE Commun. Mag..

[4] Shi W., Cao J., Zhang Q., Li Y., Xu L. (2016). Edge Computing: Vision and Challenges. IEEE Internet Things J..

[5] Taleb T., Dutta S., Ksentini A., Lqbal M., Flinck H. (2017). Mobile edge computing potential in making cities smarter. IEEE Commun. Mag..

[6] Xu Y., Chen X., Liu A., Hu C.A. (2017). Latency and Coverage Optimized Data Collection Scheme for Smart Cities Based on Vehicular Ad-hoc Networks. Sensors.

[7] Curiac D.I. (2016). Towards wireless sensor, actuator and robot networks: Conceptual framework, challenges and perspectives. J. Netw. Comp. Appl..

[8] Alves M., Pirmez L., Rossetto S., Delicato F., de Farias C., Pires P., dosSantos I., Zomaya A. (2017). Damage prediction for wind turbines using wireless sensor and actuator networks. J. Netw. Comp. Appl..

[9] Kan Y., Akerberg J., Gidlund M., Bjorkman M. Realization and measurements of industrial wireless sensor and actuator networks. Proceedings of the 2015 IEEE International Conference on Automation Science and Engineering.

[10] Sales N., Remedios O., Arsenio A. Wireless sensor and actuator system for smart irrigation on the cloud. Proceedings of the 2nd IEEE World Forum on Internet of Things.

[11] Zhou J., Chungui L., Zhang Z. Intelligent transportation system based on SIP/ZigBee architecture. Proceedings of the 2011 International Conference on Image Analysis and Signal Processing.

[12] Celtek S.A., Soy H. An application of building automation system based on wireless sensor/actuator networks. Proceedings of the 9th International Conference on Application of Information and Communication Technologies.

[13] Ghatak S., Bose S., Roy S. Intelligent wall mounted wireless fencing system using wireless sensor actuator network. Proceedings of the 2014 International Conference on Computer Communication and Informatics (ICCCI 2014).

[14] Kułakowski P., Calle E., Marzo J.L. (2013). Performance study of wireless sensor and actuator networks in forest fire scenarios. Int. J. Commun. Syst..

[15] Tang J., Liu A., Zhao M., Wang T. (2018). An Aggregate Signature Based Trust Routing for Data Gathering in Sensor Networks. Secur. Commun. Netw..

[16] Chen X., Xu Y., Liu A. (2017). Cross Layer Design for Optimizing Transmission Reliability, Energy Efficiency, and Lifetime in Body Sensor Networks. Sensors.

[17] Ma F., Liu X., Liu A., Zhao M., Huang C., Wang T. (2018). A Time and Location Correlation Incentive Scheme for Deeply Data Gathering in Crowdsourcing Networks. Wirel. Commun. Mob. Comput..

[18] Gonizzi P., Ferrari G., Gay V., Laguay J. (2015). Data dissemination scheme for distributed storage for IoT observation systems at large scale. Inf. Fusion.

[19] Maia G., Guidoni D., Viana A., Aquino A., Mini R., Loureiro A. (2013). A distributed data storage protocol for heterogeneous wireless sensor networks with mobile sinks. Ad Hoc Netw..

[20] Shao M., Zhu S., Zhang W., Cao G., Yang Y. (2009). pDCS: Security and privacy support for data-centric sensor networks. IEEE Trans. Mob. Comput..

[21] Ren Y., Oleshchuk V., Li F. (2013). Optimized secure and reliable distributed data storage scheme and performance evaluation in unattended WSNs. Comput. Commun..

[22] Talari A., Rahnavard N. (2016). CStorage: Decentralized compressive data storage in wireless sensor networks. Ad Hoc Netw..

[23] Albano M., Chessa S. (2015). Replication vs. Erasure coding in data centric storage for wireless sensor networks. Comput. Netw..

[24] Shen H., Zhao L., Li Z. (2011). A distributed spatial-temporal similarity data storage scheme in wireless sensor networks. IEEE Trans. Mob. Comput..

[25] Yu Z., Xiao B., Zhou S. (2010). Achieving optimal data storage position in wireless sensor networks. Comput. Commun..

[26] Ma X., Gao J., Wang W., Wang J. (2012). A virtual-ring-based data storage and retrieval scheme in wireless sensor networks. Int. J. Distrib. Sens. Netw..

[27] Liu X., Huang Q., Zhang Y. (2007). Balancing push and pull for efficient information discovery in large-scale sensor networks. IEEE Trans. Mob. Comput..

[28] Lin C., Kuo J., Liu B., Tsai M. (2012). GPS-free, boundary-recognition-free, and reliable double-ruling-based information brokerage scheme in wireless sensor networks. IEEE Trans. Comput..

[29] Liang J., Wang J., Li T., Chen J. (2010). Maximum lifetime algorithm for precise data gathering based on tree in wireless sensor networks. Chin. J. Softw..

[30] Liu C., Wu K., Pei J. (2007). An energy-efficient data collection framework for wireless sensor networks by exploiting spatiotemporal correlation. IEEE Trans. Parallel Distrib. Syst..

[31] Dong M., Ota K., Liu A., Guo M. (2016). Joint Optimization of Lifetime and Transport Delay under Reliability Constraint Wireless Sensor Networks. IEEE Trans. Parallel Distrib. Syst..

[32] Jiang H., Jin S., Wang C. (2011). Prediction or Not? An energy-efficient framework for clustering-based data collection in wireless sensor networks. IEEE Trans. Parallel Distrib. Syst..

[33] Liu Y., Dong M., Ota K., Liu A., Guo M. (2016). ActiveTrust: Secure and Trustable Routing in Wireless Sensor Networks. IEEE Trans. Inf. Forensics Secur..

[34] Kumar A., Sivalingam K., Kumar A. (2013). On reducing delay in mobile data collection based wireless sensor networks. Wirel. Netw..

[35] Francesco M., Das S., Anastasi G. (2011). Data collection in wireless sensor networks with mobile elements: A Survey. ACM Trans. Sens. Netw..

[36] Gao S., Zhang H., Das S. (2011). Efficient data collection in wireless sensor networks with path-constrained mobile sinks. IEEE Trans. Mob. Comput..

[37] Khan A., Abdullah A., Anisi M., Bangash J. (2014). A comprehensive study of data collection schemes using mobile sinks in wireless sensor networks. Sensors.

[38] Dhamdhere S., Guru S. (2014). Robust data collection in wireless sensor networks with mobile sinks. Int. J. Comput. Sci. Inf. Technol..

[39] Yang S., Adeel U., Tahir Y., Mccann J. (2017). Practical opportunistic data collection in wireless sensor networks with mobile sinks. IEEE Trans. Mob. Comput..

[40] Zhang X., Dai H., Xu L., Chen G. (2013). Mobility-assisted data-gathering strategies in WSNs. Chin. J. Softw..

[41] Sugihara R., Gupta R. (2011). Path planning of data mules in sensor networks. ACM Trans. Sens. Netw..

[42] Cuevas Á., Urueña M., Romeral R., Larrabeiti D. (2010). Data centric storage technologies: Analysis and enhancement. Sensors.

[43] Cuevas Á., Urueña M., Cuevas R., Romeral R. (2011). Modelling data-aggregation in multi-replication data centric storage systems for wireless sensor and actuator Networks. IET Commun..

[44] Cuevas Á., Urueña M., Veciana G., Cuevas R., Crespi N. (2014). Dynamic data-centric storage for long-term storage in wireless sensor and actuator networks. Wirel. Netw..

[45] Ngai E., Zhou Y., Lyu M., Liu J. (2010). A delay-aware reliable event reporting framework for wireless sensor–actuator networks. Ad Hoc Netw..

[46] Karimi H., Medhati O., Zabolzadeh H., Jamalpoor A. (2015). Implementing a reliable, fault tolerance and secure framework in the wireless sensor-actuator networks for events reporting. Proc. Comput. Sci..

[47] Mustafa A., Turgut D. (2013). Lightweight routing with dynamic interests in wireless sensor and actuator networks. Ad Hoc Netw..

[48] Shen H., Li Z. (2016). A Kautz-based wireless sensor and actuator network for real-time, fault-tolerant and energy-efficient transmission. IEEE Trans. Mob. Comput..

[49] Kakarla J., Majhi B., Battula R. (2015). Comparative analysis of routing protocols in wireless sensor–actuator networks: A review. Int. J. Wirel. Inf. Netw..

[50] Mo L., Cao X., Chen J., Sun Y. (2014). Collaborative estimation and actuation for wireless sensor and actuator networks. IFAC Proc. Vol..

[51] Zeng Y., Li D., Vasilakos A. (2013). Real-time data report and task execution in wireless sensor and actuator networks using self-aware mobile actuators. Comput. Commun..

[52] Konstantopoulos C., Pantziou G., Venetis I., Gavalas D. (2016). Efficient event handling in wireless sensor and actuator networks: An on-line computation approach. J. Netw. Comput. Appl..

[53] Yi J., Shi W., Tang Y., Xu L. (2010). A dynamic task scheduling for wireless sensor and actuator networks. Chin. J. Electron..

[54] Xu Z., Chen C., Guo Y., Guan X. (2011). Ballooning: An agent-based search strategy in wireless sensor and actuator networks. IEEE Commun. Lett..

[55] Xu Z., Chen C., Cheng B., Guan X. (2012). Sharing mobility strategy improves location service in wireless sensor and actuator networks. IEEE Commun. Lett..

[56] Bettstetter C., Hartenstein H., Pérezcosta X. (2004). Stochastic properties of the random waypoint mobility model. Wirel. Netw..

[57] Bose P., Morin P., Stojmenovi’c I., Urrutia J. (2001). Routing with guaranteed delivery in ad hoc wireless networks. Wirel. Netw..

[58] Li J., Liu X. (2013). An important aspect of big data: Data usability. J. Comput. Res. Dev..

[59] Ota K., Dong M., Gui J., Liu A. (2018). QUOIN: Incentive Mechanisms for Crowd Sensing Networks. IEEE Netw. Mag..

